# Supplementary material for: The Myosin-V Myo51 and Alpha-Actinin Ain1p Cooperate during Contractile Ring Assembly and Disassembly in Fission Yeast Cytokinesis
Source: J Fungi (Basel). 2024 Sep 12;10(9):647. doi: 10.3390/jof10090647 (PMC11433389; doi:10.3390/jof10090647)
Supplement: Supplementary file 1 [file jof-10-00647-s001.zip › Supplemental Table S1.pdf]

Table S1. Strains list

Strain	Genotype	Reference/Origin
SMLM		
IRT206	*h- cdc15-mEos3.2-kanMX6 cdc25-22 ade6-M210 ura4-D18*	Laplante et al., 2016
IRT207	*h+ myo2-mEos3.2-kanMX6 cdc25-22 ade6-M21X leu1-32 ura4-D18*	Laplante et al., 2016
CL259	*h+ kanMX6-Prng2-mEos3.2-rng2 cdc25-22 ade6-M21X ura4-D18*	Laplante et al., 2016
Confocal		
IRT69	*h- kanMX6-Prng2-mEGFP-rng2 sad1-RFP-kanMX6 ade6-M21X leu1-32 ura-D18*	Tebbs et al., 2013
CL345	*h+ myo51-3GFP-kanMX6 kanMX6-Pmyp2short-mCherry-myp2*	This work
CL459	*h- cdc12-3GFP-kanMX6 kanMX6-Pmyp2short-mCherry-myp2*	This work
CL520	*kanMX6-Pcdc15-mEGFP-cdc15 Pmyo2short-mCherry-myo2-kanMX6*	This work
CL563	*h+ kanMX6-Pmyo2short-mEGFP-myo2 sad1-RFP-kanMX6*	Bellingham-Johnstun et al., 2021
CL770	*h+ imp2-mEGFP-kanMX6 ade6-M216 his3-D1 leu1-32 ura4-D18*	Bellingham-Johnstun et al., 2022
CL834	*kanMX6-Pmyp2short-mCherry-myp2 kanMX6-Pmyo2short-mEGFP-myo2 ade6-M21X his3-D1 leu1-32 ura4-D18*	This work
CL1060	*h+ Δain1::natMX6 kanMX6-Pmyo2short-mEGFP-myo2 sad1-RFP-kanMX6*	This work
CL1142	*h+ Δmyo51::kanMX6 kanMX6-Pmyo2short-mEGFP-myo2 sad1-RFP-kanMX6*	This work
CL1157	*myo51-3GFP-kanMX6 cdc15-mCherry-kanMX6*	This work
CL1174	*Δain1::natMX6 Δmyo51::kanMX6 kanMX6-Pmyo2short-mEGFP-myo2 sad1-RFP-kanMX6*	This work
CL1187	*Δain1::natMX6 Δmyp2::kanMX6 kanMX6-Pmyo2short-mEGFP-myo2 sad1-RFP-kanMX6*	This work
