# Supplementary material for: The Myosin-V Myo51 and Alpha-Actinin Ain1p Cooperate during Contractile Ring Assembly and Disassembly in Fission Yeast Cytokinesis
Source: J Fungi (Basel). 2024 Sep 12;10(9):647. doi: 10.3390/jof10090647 (PMC11433389; doi:10.3390/jof10090647)
Supplement: Supplementary file 1 [file jof-10-00647-s001.zip › Supplemental Table S2.pdf]

# Table S2. Summary statistics of swarm plots

| Analysis type | Genotype | Mean | StDev | n= |
|---|---|---|---|---|
| Clumping duration (min), Figure 1E | *Δain1* | 19.7 | 7.2 | 45 |
| | *Δain1 Δmyo51* | 20.0 | 9.9 | 26 |
| | *Δain1 Δmyp2* | 26.5 | 6.6 | 12 |
| Clumping fluoresence intensity (A.U.), Figure 1G | *Δain1* | $1.9x10^4$ | $8.4x10^3$ | 19 |
| | *Δain1 Δmyo51* | $1.3x10^4$ | $4.4x10^3$ | 19 |
| % constricted at start of shedding, Figure 3C | Wild type | 61.8 | 6.0 | 38 |
| | *Δain1* | 58.0 | 7.9 | 44 |
| | *Δmyo51* | 65.0 | 5.0 | 42 |
| | *Δain1 Δmyo51* | 50.6 | 8.5 | 51 |
| Shedding duration (min), Figure 3D | Wild type | 13.8 | 3.6 | 30 |
| | *Δain1* | 14.8 | 3.6 | 30 |
| | *Δmyo51* | 13.7 | 3.6 | 30 |
| | *Δain1 Δmyo51* | 23.7 | 5.8 | 30 |
| Timing of ring assembly (min), Figure S1A | Wild type | 17.0 | 3.5 | 43 |
| | *Δain1* | 12.7 | 2.4 | 38 |
| | *Δmyo51* | 30.2 | 4.7 | 47 |
| | *Δain1 Δmyo51* | 21.4 | 4.1 | 46 |
| Timing of constriction onset (min), Figure S1B | Wild type | 36.6 | 3.7 | 37 |
| | *Δain1* | 37.9 | 4.6 | 34 |
| | *Δmyo51* | 41.8 | 3.8 | 41 |
| | *Δain1 Δmyo51* | 43.8 | 4.2 | 42 |
| Timing of clumping onset (min), Figure S1C | *Δain1* | 9.8 | 6.0 | 25 |
| | *Δain1 Δmyo51* | 13.5 | 5.0 | 26 |
| Timing of clumping end (min), Figure S1D | *Δain1* | 29.4 | 9.1 | 25 |
| | *Δain1 Δmyo51* | 33.5 | 8.7 | 26 |
| Timing of shedding onset (min), Figure S1E | Wild type | 64.1 | 4.3 | 43 |
| | *Δain1* | 65.5 | 5.7 | 34 |
| | *Δmyo51* | 72.0 | 5.3 | 29 |
| | *Δain1 Δmyo51* | 70.2 | 6.0 | 37 |
| Timing of ring disassembly (min), Figure S1F | Wild type | 77.0 | 7.0 | 24 |
| | *Δain1* | 79.2 | 4.5 | 19 |
| | *Δmyo51* | 82.8 | 4.3 | 11 |
| | *Δain1 Δmyo51* | 89.1 | 6.6 | 13 |
| Maturation duration (min), Figure S1G | Wild type | 19.4 | 5.4 | 37 |
| | *Δain1* | 25.2 | 4.8 | 34 |
| | *Δmyo51* | 11.9 | 4.8 | 41 |
| | *Δain1 Δmyo51* | 22.2 | 4.5 | 41 |
| Constriction rate (μm/min), Figure S1H | Wild type | 0.28 | 0.04 | 15 |
| | *Δain1* | 0.27 | 0.03 | 20 |
| | *Δmyo51* | 0.31 | 0.04 | 15 |
| | *Δain1 Δmyo51* | 0.28 | 0.04 | 20 |
| Width of shed (% of cell width), Figure S1J | Wild type | 77.8 | 10.2 | 10 |
| | *Δain1* | 78.0 | 6.3 | 10 |
| | *Δmyo51* | 74.4 | 11.4 | 10 |
| | *Δain1 Δmyo51* | 89.4 | 5.8 | 10 |
| Global fluoresecence intensity of DMSO and LatA (A.U.), Figure S2 | DMSO | $1.5x10^7$ | $1.4x10^6$ | 15 |
| | LatA | $1.5x10^7$ | $1.8x10^6$ | 15 |
