# Supplementary material for: The Myosin-V Myo51 and Alpha-Actinin Ain1p Cooperate during Contractile Ring Assembly and Disassembly in Fission Yeast Cytokinesis
Source: J Fungi (Basel). 2024 Sep 12;10(9):647. doi: 10.3390/jof10090647 (PMC11433389; doi:10.3390/jof10090647)
Supplement: Supplementary file 1 [file jof-10-00647-s001.zip › Supplemental Table S3.pdf]

Table S3. Results of Log-rank tests of outcomes plots

Figure 1C. Assembly completion

	Wild type	*Δain1*	*Δmyo51*	*Δain1 Δmyo51*
Wild type	-	$p < 0.001$	$p < 0.001$	$p < 0.001$
Δain1	$p < 0.001$	-	$p < 0.001$	$p < 0.001$
Δmyo51	$p < 0.001$	$p < 0.001$	-	$p < 0.001$
Δain1 Δmyo51	$p < 0.001$	$p < 0.001$	$p < 0.001$	-

Figure 1C. Onset of constriction

	Wild type	*Δain1*	*Δmyo51*	*Δain1 Δmyo51*
Wild type	-	$p=0.1333$	$p < 0.001$	$p < 0.001$
Δain1	$p=0.1333$	-	$p < 0.05$	$p < 0.001$
Δmyo51	$p < 0.001$	$p < 0.05$	-	$p < 0.05$
Δain1 Δmyo51	$p < 0.001$	$p < 0.001$	$p < 0.05$	-

Figure 1C. Clumping start

	Δain1	*Δain1 Δmyo51*
Δain1	-	$p=0.1260$
Δain1 Δmyo51	$p=0.1260$	-

Figure 1C. Clumping end

	Δain1	*Δain1 Δmyo51*
Δain1	-	$p=0.1333$
Δain1 Δmyo51	$p=0.1333$	-

Figure 3E. Shedding start

	Wild type	*Δain1*	*Δmyo51*	*Δain1 Δmyo51*
Wild type	-	$p=0.1855$	$p < 0.001$	$p < 0.001$
Δain1	$p=0.1855$	-	$p < 0.05$	$p < 0.05$
Δmyo51	$p < 0.001$	$p < 0.05$	-	$p=0.4025$
Δain1 Δmyo51	$p < 0.001$	$p < 0.05$	$p=0.4025$	-

Figure 3E. Disassembly completion

	Wild type	*Δain1*	*Δmyo51*	*Δain1 Δmyo51*
Wild type	-	$p=0.7043$	$p=0.1531$	$p < 0.05$
Δain1	$p=0.7043$	-	$p < 0.05$	$p < 0.001$
Δmyo51	$p=0.1531$	$p < 0.05$	-	$p < 0.05$
Δain1 Δmyo51	$p < 0.05$	$p < 0.001$	$p < 0.05$	-
