# Supplementary figures and images for: The Myosin-V Myo51 and Alpha-Actinin Ain1p Cooperate during Contractile Ring Assembly and Disassembly in Fission Yeast Cytokinesis
Source: J Fungi (Basel). 2024 Sep 12;10(9):647. doi: 10.3390/jof10090647 (PMC11433389; doi:10.3390/jof10090647)

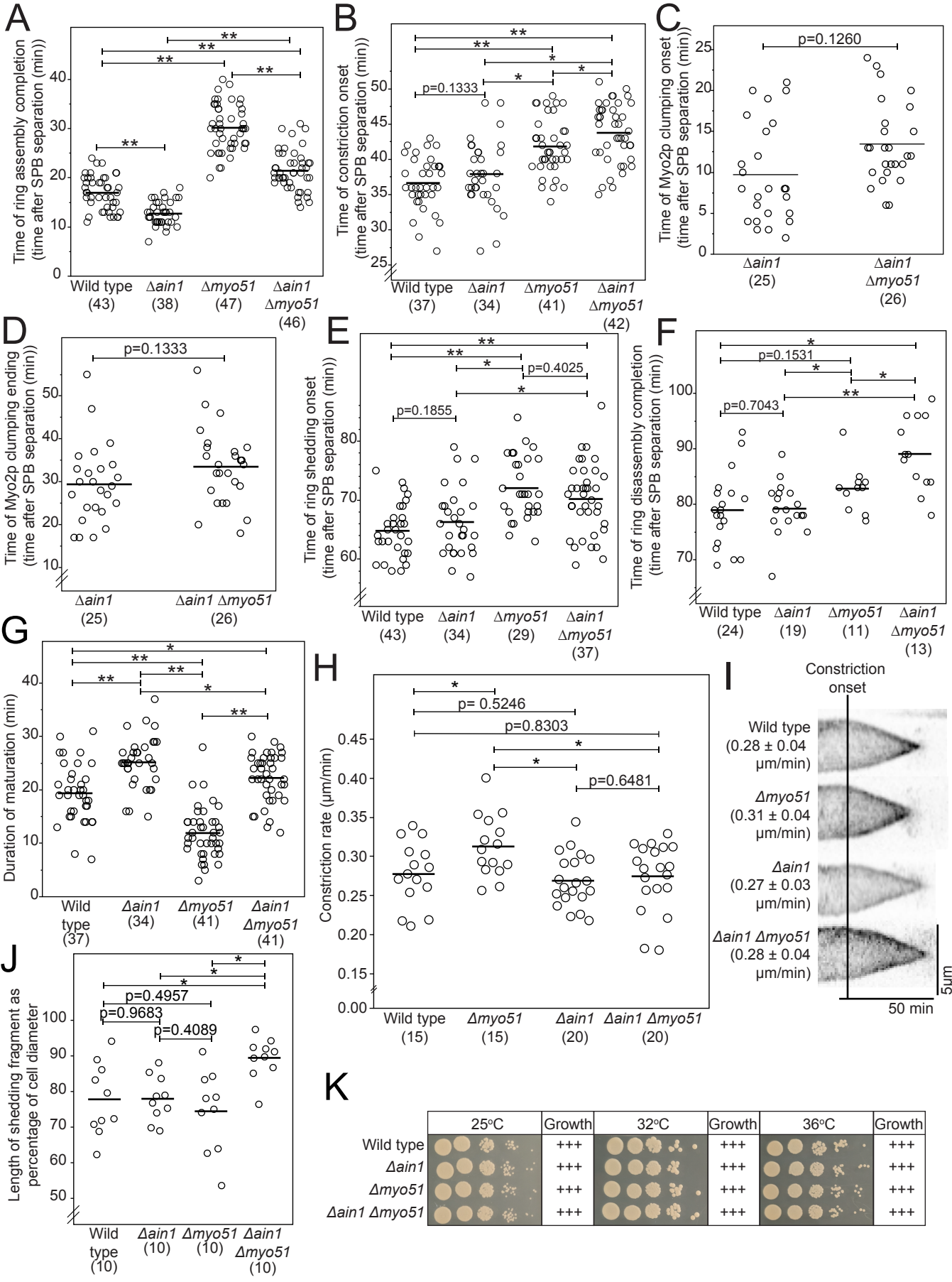

Supplement: Supplementary file 1 [file jof-10-00647-s001.zip › Supplemental Figure S1.pdf]

Global cytoplasmic fluorescence intensity ( $\times 10^7$ , A.U.)

$p=0.5202$

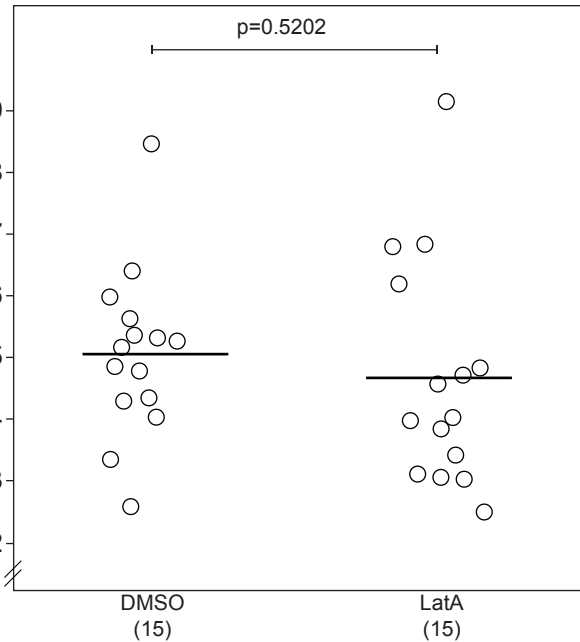

Supplement: Supplementary file 1 [file jof-10-00647-s001.zip › Supplemental Figure S2.pdf]
